# Investigating the relationships between headache characteristics and physical activity, autonomic function, psychological status, and quality of life in individuals with tension-type headache

**DOI:** 10.1186/s12883-025-04616-4

**Published:** 2026-01-07

**Authors:** Mesut Arslan, Sefa Haktan Hatık

**Affiliations:** 1 Department of Physiotherapy and Rehabilitation, Faculty of Health Sciences, Bitlis Eren University, Bitlis, Türkiye; 2 Department of Health Care Services, Türkeli Vocational School, Sinop University, Sinop, Türkiye

**Keywords:** Tension-type headache, Physical activity, Autonomic function, Psychological state, Quality of life

## Abstract

**Background:**

Tension-type headache (TTH) is a highly prevalent primary headache disorder, yet its multidimensional associations remain incompletely understood. The aim of this study was to investigate the relationship between the characteristics of headaches in individuals with Tension-Type Headache (TTH) and physical activity, autonomic function, psychological state, and quality of life.

**Methods:**

This observational descriptive study included 93 individuals aged 18–65 years with TTH (mean age 25.6 years, 84.9% female), diagnosed by a neurologist in accordance with the International Classification of Headache Disorders (ICHD-3) criteria. Headache characteristics were recorded; physical activity was assessed using the International Physical Activity Questionnaire (IPAQ-7), autonomic function was measured using the Polar Verity Sense device to assess heart rate variability (RMSSD, LF/HF), psychological status was evaluated using the Depression Anxiety Stress Scale (DASS-21), and quality of life was assessed using the Headache Impact Scale. Relationships were analyzed using Spearman correlation analysis.

**Results:**

The vast majority of individuals were inactive (33.3%) or minimally active (57%). Moderate to weak positive correlations were found between headache frequency and severity (resting, activity, and nighttime) and depression, anxiety, and stress scores (all *p* < 0.05). Additionally, headache frequency and severity were significantly associated with impaired quality of life (*p* < 0.01). In terms of autonomic function, longer headache duration and higher resting pain intensity were negatively correlated with the RMSSD value, an indicator of lower parasympathetic activity (both rho=-0.24, *p* = 0.018). A weak positive relationship was observed between headache onset time and physical activity (rho = 0.26, *p* = 0.011).,

**Conclusion:**

In this predominantly young adult and female cohort, TTH has been shown to be a multidimensional condition with weak to moderate associations to low physical activity, potentially decreased parasympathetic tone, psychological distress, and impaired quality of life. These findings map a cluster of biopsychosocial correlates in this demographic context and support the rationale for future research into integrating non-pharmacological, holistic approaches targeting physical activity, psychological support, and autonomic balance in the management of TTH.

**Trial registration:**

This crosssectional study has a design that does not require a clinical research record.

## Introduction

Tension-type headache (TTH) is characterized by mild to moderate pain that is typically bilateral, pressing, and constricting in nature [1]. It is the most common primary headache and has a lifetime prevalence of 26.1–45% in the general population [2]. It can be classified as episodic (less than 15 headache days per month) or chronic (more than 15 headache days per month) [3]. Increased pericranial myofascial sensitivity, a lower pain threshold, and an increase in myofascial trigger points have been observed in TTH [4]. However, it is not yet possible to discuss a clear etiology and pathophysiology [1].

It is stated that TTH does not change with physical activity [1]. However, a review of the literature reveals conflicting results [2, 5]. A study by González de la Flor et al. reported no significant relationship between pain intensity, duration, and frequency and physical activity in individuals with chronic TTH [2]. Another study showed a negative correlation between TTH severity and physical activity [5].

Tension-type headache is known to be associated with autonomic nervous system dysfunction [6]. Impaired vagal activity results in increased pro-inflammatory cytokines and inflammation [7]. These inflammatory processes may cause prolonged and continuous stimulation of nociceptive neurons, leading to chronic headache [8]. A relationship has also been reported between low heart rate variability and chronic pain. Stimulation of the ventral periaqueductal gray region has been shown to increase parasympathetic activity and heart rate variability and reduce pain in individuals with chronic pain [9]. A meta-analysis study also found lower heart rate variability in individuals with headaches [8]. However, no study has been found on the relationship between TTH and autonomic function levels.

Psychological stress is an important trigger for TTH. This stress can lead to involuntary muscle activation, and chronic muscle activation can also cause pain [10]. Indeed, a meta-analysis study found that individuals with tension-type headache had lower pressure pain thresholds in the trigeminal and neck regions [11]. Furthermore, depression and anxiety have been reported to be more prevalent in individuals with TTH compared to healthy controls [12]. While several studies have established a clear association between TTH and psychological comorbidities such as depression and anxiety [12, 13] comprehensive investigations simultaneously examining the interplay of headache characteristics with psychological status, autonomic function, physical activity, and quality of life remain limited. TTH affects the individual’s daily activities, family, and social relationships, which increases the individual’s stress levels and reduces their cognitive capacity and sleep quality. All these conditions can significantly reduce an individual’s quality of life [2]. However, only one study has been found on the relationship between TTH and quality of life. This study also reported that there was no significant relationship between pain intensity, duration, and frequency and quality of life in individuals with chronic TTH [2].

Tension-type headache (TTH) is the most common primary headache, yet its pathophysiology remains poorly understood. The literature on the relationship between physical activity and TTH contains conflicting findings; moreover, there are very few studies that comprehensively examine the relationship between headache characteristics and autonomic function, psychological state, and quality of life. Therefore, this study aimed to examine the relationship between headache characteristics (intensity, duration, frequency) and physical activity, autonomic function, psychological state, and quality of life in individuals with TTH.

The central hypothesis of this study is that the clinical characteristics of TTH are associated with multiple physiological and psychosocial parameters. Specifically, it was postulated that headache intensity, frequency, and duration would demonstrate meaningful associations with physical activity levels, autonomic function indices (RMSSD, LF/HF), psychological status (depression, anxiety, stress), and quality of life. Accordingly, the alternative hypothesis (H1) posits that TTH characteristics exhibit significant relationships with these variables, whereas the null hypothesis (H0) asserts the absence of any statistically significant associations.

## Method

### Design of the study

This research is an observational descriptive study.

### Subjects

This observational descriptive study was conducted between June 2025 and September 2025, and all participants were recruited from the Neurology Outpatient Clinics of Bitlis State Hospital and Tatvan State Hospital. All individuals were diagnosed with TTH by neurologists in accordance with the International Classification of Headache Disorders (ICHD-3) criteria. During the diagnostic process, each participant underwent a standard neurological examination, detailed medical and headache history was obtained, red-flag symptoms were screened, and when clinically indicated, brain imaging (CT/MRI) or relevant laboratory tests were performed to exclude secondary headache causes. Only individuals who fully met the ICHD-3 criteria and whose diagnoses were clinically confirmed by neurologists were included in the study. A total of 107 individuals were assessed for eligibility, of whom 14 did not meet the inclusion criteria and were consequently excluded; thus, 93 participants aged 18–65 years who met the diagnostic criteria were included in the final analysis. Exclusion criteria were: serious pathologies (neurological (MS, Parkinson’s, etc.), rheumatological, cardiovascular, psychiatric diseases), history of trauma/surgery to the head and neck region, history of malignancy, pregnancy, other primary and secondary headache types, and individuals who were uncooperative [14, 15]. The study was conducted in accordance with the Declaration of Helsinki and Good Clinical Practice guidelines. The study commenced after obtaining the necessary ethical committee approval. Participants were informed and consent was obtained in accordance with the informed consent form.

### Measures

The primary outcome of this study was to assess the relationship between headache characteristics and both physical activity levels and autonomic function (HRV – RMSSD/LF–HF). The secondary outcomes involved examining the associations between headache characteristics and psychological status (depression, anxiety, stress) as well as quality of life. The sample size was determined based on correlation coefficients previously reported in the literature regarding the relationship between physical activity and headache characteristics, ensuring alignment with the primary outcome of the study.

Demographic characteristics including age, sex, body mass index (BMI), and lifestyle factors such as tobacco and alcohol consumption were recorded for all participants. Headache characteristics were evaluated, including onset time, frequency (days per month), duration, and intensity assessed using the Numeric Pain Scale (NPS), a self-reported 0–10 scale where “0” indicates no pain and “10” represents unbearable pain [16].

Individuals’ physical activity levels were assessed using the International Physical Activity Questionnaire-7 (IPAQ-7), which has validity and reliability in Turkish [17]. This questionnaire contains a total of 7 questions. The total score is calculated based on the duration (minutes) and frequency (days) of walking, moderate-intensity, and vigorous activities performed for at least 10 min at a time. Based on the total score, individuals’ physical activity levels were classified as inactive (< 600 MET-min/week), minimally active (600–3000 MET-min/week), and highly active (> 3000 MET-min/week) [17].

Autonomic functions were assessed using heart rate variability. Heart rate variability was measured for 1 min using the non-invasive Polar Verity Sense device (Polar Electro Oy, Professorintie 5, FI-90440 Kempele, Finland), and the obtained data were transferred to the KubiosHRV mobile application. The parameters RMSSD (root mean square of successive N-N intervals) and LF/HF (low frequency/high frequency) ratio, which indicate heart rate variability, were recorded [18]. The use of this ultra-short-term recording protocol is supported for the estimation of time-domain parameters, specifically RMSSD as a marker of parasympathetic activity [19]. During the measurement, patients were seated in a comfortable chair and instructed not to talk or move. Patients were asked to refrain from smoking and drinking coffee for at least 2 h prior to the autonomic measurement and from consuming alcohol for 12 h prior. Measurements were taken at the same time of day for the same individuals. Patients were instructed to eat a light meal 2–4 h before the measurement and to get a normal night’s sleep the night before the measurement. Given the novel application of this protocol in our TTH cohort and the cross-sectional design, the autonomic findings are presented as exploratory and hypothesis-generating.

Individuals’ psychological states were assessed using the Depression Anxiety Stress Scale (DASS21), which has been adapted into Turkish [20]. The scale inquires about the individual’s state over the past week. It is a 21-item scale consisting of three subscales: depression, anxiety, and stress. All subscale scores range from 0 to 21. High scores on the questionnaire indicate the presence and severity of depression, anxiety, and stress [20].

Finally, individuals’ quality of life was assessed using the Headache Impact Scale, which has established validity and reliability in Turkish [21]. It is a 6-item measure that assesses vitality, pain, psychological state, social, role, and cognitive function. Each item is answered using a 5-point Likert scale (6 = never, 8 = rarely, 10 = occasionally, 11 = very often, 13 = always). The total scale score ranges from 36 to 78; high scores indicate a higher level of impact on the individual’s quality of life and are evaluated in four groups: ≤49 points (no impact), 50–55 points (some impact), 56–59 points (significant impact), and ≥ 60 points (severe impact) [21].

### Sample size

The required sample size for the study was calculated using the G*Power software package (G*Power Ver. 3.0.10, Franz Faul, University of Kiel, Germany). In a similar study in the literature on this topic, calculations using the correlation coefficient between physical activity and headache frequency showed that 83 participants were needed to achieve an effect size of 0.27 and 80% power at α = 0.05 [2].

### Data analysis

Descriptive statistical data were presented using counts, percentages, and mean ± standard deviation values. The normality of the variables was assessed using the Shapiro-Wilk test. Since the variables were not normally distributed, Spearman’s correlation coefficient was used to analyze the relationship between headache characteristics and physical activity, autonomic function, psychological status, and quality of life. Correlation coefficient values were determined as 0.0–0.19.0.19 very weak, 0.20–0.39 weak, 0.40–0.59 moderate, 0.60–0.79 high, 0.80–1.00.80.00 very high [22]. IBM SPSS Statistics 21.0 (IBM Corp. Released 2012. IBM SPSS Statistics for Windows, Version 21.0. Armonk, NY: IBM Corp.) and MS-Excel 2007 software were used for statistical analyses and calculations. Statistical significance was set at *p* < 0.05.

## Results

Data from 93 individuals were analyzed in the study. The vast majority of individuals were women (79, 84.9%), with a mean age of 25.61 ± 9.65 years and a mean BMI of 24.26 ± 4.97 kg/m². The mean headache onset time was 53.24 ± 41.39 months, headache frequency was 11.69 ± 8.39 (days/month), and headache severity at rest was 6.06 ± 2.55 (NPS/0–10). Furthermore, the majority of individuals had inactive (33.3%) or minimally active (57%) physical activity levels (Table 1).

**Table 1:** Descriptive data of participants

Parameters			Mean ± Standard Deviation or Number (Percentage)
Sociodemographic Data	Age (years)		25.61 ± 9.65
	BMI (kg/m²)		24.26 ± 4.97
	Gender	Male	14 (15.1)
		Female	79 (84.9)
	Smoking	Yes	27 (29.1)
		No	66 (71.0)
	Alcohol	Yes	0 (0)
		No	93 (100)
Headache Characteristics	Headache onset time (month)		53.24 ± 41.39
	Headache Frequency (days/month)		11.69 ± 8.39
	Headache Duration (minutes)		211.73 ± 343.93
	Headache severity at rest (NPS/0–10)		6.06 ± 2.55
	Headache severity during activity (NPS/0–10)		6.54 ± 2.64
	Headache severity during the night (NPS/0–10)		5.08 ± 3.50
	Use of medication for headache	Yes	53 (57)
		No	40 (43)
Level of Physical Activity (IPAQ-7)	Inactive		31 (33.3)
	Minimally active		53 (57)
	Very active		9 (9.7)

*X* Mean, *SD* Standard Deviation, *BMI* Body Mass Index, *IPAQ* International Physical Activity Questionnaire

When examining the relationship between individuals’ headache characteristics and physical activity, autonomic function, psychological state, and quality of life (Table 2):

**Table 2:** The relationship between headache characteristics and physical activity, autonomic function, psychological state, and quality of life

Parameters		Headache Characteristics											
		Headache onset time (month)		Headache Frequency (days/month)		Headache Duration (minutes)		Headache severity at rest (NPS/0–10)		Headache severity during activity (NPS/0–10)		Headache severity during the night (NPS/0–10)	
		rho	*p*	rho	*p*	rho	*p*	rho	*p*	rho	*p*	rho	*p*
Level of Physical Activity (IPAQ-7)		0.26	0.011	−0.03	0.74	0.11	0.285	0.04	0.686	−0.01	0.924	0.05	0.598
Autonomic Functions (heart rate variability)	RMSSD	−0.12	0.237	−0.03	0.722	−0.24	0.018	−0.24	0.018	−0.16	0.12	−0.11	0,295
	LF/HF	−0.02	0.805	−0.04	0.675	0.05	0.612	−0.17	0.096	−0.08	0.434	−0.15	0,141
Psychological State (DASS21)	Depression	0.00	0.932	0.54	< 0.001	0.31	0.002	0.47	< 0.001	0.56	< 0.001	0.36	< 0,001
	Anxiety	0.12	0.237	0.32	0.001	0.08	0.412	0.21	0.036	0.35	< 0.001	0.32	0,001
	Stress	0.0	0.394	0.41	< 0.001	−0.02	0.818	0.05	0.596	0.35	0.001	0.37	< 0,001
Quality of Life (Headache Impact Score)		0.21	0.036	0.39	< 0.001	−0.00	0.958	0.18	0.075	0.29	0.004	0.33	0.001

*NPS* Numerical Pain Scale, *rho* Spearman Correlation Coefficient, *IPAQ-7* International Physical Activity Questionnaire-7, *RMSSD,* *LF/HF*, *DASS21* Depression Anxiety Stress Scale

A weak positive correlation was found between headache onset time and physical activity (rho = 0.26, *p* = 0.011) and quality of life (rho = 0.21, *p* = 0.036).

A positive moderate correlation was found between headache frequency and depression (rho = 0.54, p = < 0.001) and stress (rho = 0.41, p = < 0.001), and a positive weak correlation was found between headache frequency and anxiety (rho = 0.32, *p* = 0.001) and quality of life (rho = 0.39, p = < 0.001).

A weak negative correlation was found between headache duration and RMSSD (rho=−0.24, *p* = 0.018), and a weak positive correlation was found between headache duration and depression (rho = 0.31, *p* = 0.002).

A weak negative correlation was found between headache intensity at rest and RMSSD (rho=−0.24, *p* = 0.018), a weak positive correlation between headache intensity at rest and depression (rho = 0.47, p = < 0.001), anxiety (rho = 0.21, *p* = 0.036).

A positive moderate correlation was found between headache severity during activity and depression (rho = 0.56, p = < 0.001); anxiety (rho = 0.35, p = < 0.001), stress (rho = 0.35, *p* = 0.001), and quality of life (rho = 0.29, *p* = 0.004).

Nighttime headache intensity showed a positive weak correlation with depression (rho = 0.36, p = < 0.001), anxiety (rho = 0.32, *p* = 0.001), stress (rho = 0.37, p = < 0.001), and quality of life (rho = 0.33, *p* = 0.001) (Table 2).

## Discussion

This study aimed to examine the relationships between headache characteristics and physical activity level, psychological status, autonomic nervous system functions, and quality of life a clinical sample of individuals with TTH, predominantly comprised of young women. The findings indicated several modest but statistically significant associations. The duration of headache onset showed a weak positive association with both physical activity level and quality of life. Headache frequency demonstrated moderate positive correlations with depression and stress, and weaker positive correlations with anxiety and reduced quality of life. The weak negative correlation between headache duration and RMSSD is suggestive of a potential decrease in parasympathetic activity, while its weak positive association with depression highlights the relevance of psychological determinants. Additionally, headache intensity (evaluated during rest, activity, and nighttime) showed moderate positive correlations with depression and weaker positive correlations with anxiety, stress, and impaired quality of life. Taken together, these cross-sectional associations suggest that clinical features of TTH may be interrelated with physical activity patterns, autonomic nervous system function, psychological status, and quality of life in a multidimensional manner within this demographic context. However, the predominantly weak strength of these correlations indicates they are suggestive rather than definitive, and given the study design, they represent associations, not causal links.

Although direct evidence on the relationship between physical activity and TTH is limited, existing studies provide important clues regarding the role of physical inactivity in primary headache disorders. Various studies have reported that low levels of physical activity are associated with primary headaches, including TTH and migraine [23, 24]. A study conducted on migraine patients further showed that these individuals are generally less physically active than healthy controls, and that low physical activity is associated with increased migraine frequency [25]. Moreover, an actigraphy-based study indicated that TTH itself may negatively affect physical activity levels [5]. Taken together, these findings suggest that reduced physical activity may be linked to primary headache types, and is observed in conjunction with both the onset and persistence of TTH. Consistent with these observations, the majority of participants in our study were classified as inactive or minimally active, supporting the observed co-occurrence of physical inactivity with the clinical profile of TTH in this population.

Weak but significant negative correlation was found between headache duration and RMSSD, and between headache intensity during rest and RMSSD in our cohort. Consistent with our exploratory HRV protocol focusing on time-domain parameters, these findings are in line with previous studies suggesting decreased parasympathetic activity and autonomic imbalance in TTH. Indeed, Gevirtz has stated that both peripheral muscle mechanisms and sympathovagal imbalance may play a role in the pathophysiology of TTH [10]. Similarly, Tumurbaatar and colleagues showed that there is a decrease in vagal indices in TTH patients and that this relationship may be largely mediated by psychological stress factors [6]. Furthermore, Koenig and colleagues’ meta-analysis reported that parasympathetic dysfunction is a consistent finding in chronic headache cases and that vagal activity tends to decrease in TTH [8]. Although the correlations in our study were weak, the consistency with prior research suggests that autonomic dysfunction may represent a subtle but relevant component of TTH pathophysiology. These findings also raise the possibility that changes in vagal activity may be interrelated with psychological stress and pain modulation pathways, which is consistent with a multifatorial model of autonomic imbalance in TTH rather than a primary isolated mechanism. Thus, while our exploratory analysis supports an association between reduced parasympathetic function (as indicated by RMSSD) and clinical features of TTH, the weak correlations underscore that this relationship is likely shaped by complex, indirect mechanisms. Given the cross-sectional design, these correlations cannot determine whether autonomic changes precede, follow, or co-occur with headache characteristics, but they generate hypotheses for future longitudinal investigation.

Previous evidence indicates that individuals with tension-type headache (TTH) frequently exhibit heightened anxiety and depression, which are associated with greater headache severity and impact [12, 13, 26]. These findings suggest that emotional dysregulation is commonly associated with TTH, and its correlation with headache expression could involve mechanisms such as altered pain processing and heightened stress reactivity. Our results support this possible link by showing weak but significant associations between anxiety and both headache frequency and severity, suggesting an association where anxiety is correlated with TTH symptomatology. At the same time, stress has been shown to intensify headache through sensitization mechanisms [27–29], while our findings revealed moderate associations between stress and headache frequency and weaker, though significant, associations with activity-related and nighttime pain. Notably, depressive symptoms demonstrated even stronger associations with headache frequency, duration, and severity. This aligns with longitudinal evidence suggesting that depression may contribute to TTH pathophysiology and exacerbate its clinical burden [30, 31]. Taken together, these observations highlight that anxiety, stress, and particularly depression show significant associations with the course and expression of TTH in our clinical sample, underscoring the value of incorporating psychological assessment into TTH management strategies.

Evidence from Ashina et al. indicates that TTH, particularly in its chronic form, is associated with marked reductions in physical and mental health functioning, with even greater impairment observed when migraine is comorbid [32]. Likewise, Pan et al. reported that chronic TTH substantially diminishes quality of life and contributes to increased utilization of healthcare resources [33]. Collectively, these observations underscore that TTH imposes a considerable functional and psychosocial burden, contradicting its frequent characterization as a relatively “mild” primary headache disorder. Rather, its impact appears to be shaped not solely by pain intensity but also by chronicity and its interaction with psychological and behavioral processes. Consistent with this framework, our study identified weak yet significant associations between quality of life and headache onset, frequency, and severity, with headache intensity during activity and nighttime demonstrating comparatively stronger relationships. These findings are consistent with the notion that TTH is associated with adverse effects on daily functioning through multidimensional pathways in the studied population and highlight a potential rationale for exploring management strategies that extend beyond symptomatic relief to encompass broader quality-of-life outcomes.

The study was conducted on individuals diagnosed according to ICHD-3 criteria and has a reliable sample in terms of diagnostic accuracy. The detailed determination of exclusion criteria (neurological, cardiovascular, rheumatological, and psychiatric diseases, history of trauma, etc.) contributed to the inclusion of a homogeneous group in the study and the reduction of bias. The combined and comprehensive assessment of physical activity, psychological status, quality of life, and autonomic nervous system functions provides comprehensive data on the multidimensional nature of TTH. The objective assessment of autonomic nervous system function is an important methodological strength that increases the study’s evidence-based physiological foundation.

This study has several limitations that should be considered when interpreting the findings. First, its cross-sectional design precludes any causal inferences regarding the observed associations. Therefore, the identified relationships are strictly associative and should be interpreted as hypothesis-generating rather than evidence of causal pathways. Second, the generalizability of our findings is limited by the demographic profile of our sample, which was young (mean age 25.6 years) and predominantly female (84.9%). Age and sex are known to influence autonomic function, psychological reporting, physical activity levels, and quality-of-life perceptions. Therefore, the observed associations may not fully represent older populations or men with TTH, and our results should be interpreted within this specific demographic context. Third, reliance on self-reported questionnaires for measuring physical activity, psychological state, and quality of life introduces the possibility of recall bias and subjective reporting inaccuracies. Fourth, the single-center recruitment strategy may restrict the applicability of the findings to broader or more diverse populations. Finally, while medication use for headache was recorded, it was documented only as a binary (yes/no) variable. Clinically important details such as medication type, dosage, frequency, and treatment response were not assessed, which precludes meaningful subgroup analyses and cautions against interpreting non-use as an indicator of milder headache. Most importantly, the predominantly weak magnitude of the significant correlations, while statistically valid, suggests that the observed relationships, though indicative of potential links, should be interpreted with caution and are likely mediated by a network of other unmeasured biopsychosocial factors.

## Conclusion

This cross-sectional study identifies a pattern of significant associations between TTH and multiple biopsychosocial factors, including psychological state, quality of life, autonomic function, and physical activity levels in a predominantly young adult and female clinical sample. The findings document modest but significant correlations between TTH and higher levels of depression, anxiety, and stress, as well as links to lower physical activity and reduced quality of life.

In addition, our exploratory analysis revealed weak associations with reduced parasympathetic activity (RMSSD), which are consistent with the idea of autonomic modulation being involved in the clinical expression of TTH. Taken together, these observed cross-sectional associations are consistent with a model in which TTH exists within a complex biopsychosocial context.

These interconnected findings provide a strong empirical basis for future hypothesis-driven research in broader and more diverse TTH populations. They highlight the potential value of exploring holistic approaches in TTH management that target not only symptomatic pain treatment but also consider physical activity, psychological well-being, stress management, and quality of life. Ultimately, longitudinal and interventional studies in demographically varied samples are required to clarify the directionality and mechanistic nature of these relationships before such associative findings can be translated into effective clinical strategies.

## Data Availability

All data generated or analyzed during this study are included in this published article [and its supplementary information files. Data are available from the corresponding authors upon reasonable request.

## Abbreviations

TTH: Tension-Type Headache
ICHD-3: The International Classification of Headache Disorders
NPS: Numerical Pain Scale
RHO: Spearman Correlation Coefficient
IPAQ-7: International Physical Activity Questionnaire-7
RMSSD: Root mean square of successive RR interval differences
LF/HF: Ratio Of Low-Frequency -To- High-Frequency Power
DASS21: Depression Anxiety Stress Scale-21
BMI: Body Mass Index
